# Supplementary figures and images for: Changes in intentional binding effect during a novel perceptual-motor task
Source: PeerJ. 2018 Dec 11;6:e6066. doi: 10.7717/peerj.6066 (PMC6294047; doi:10.7717/peerj.6066)

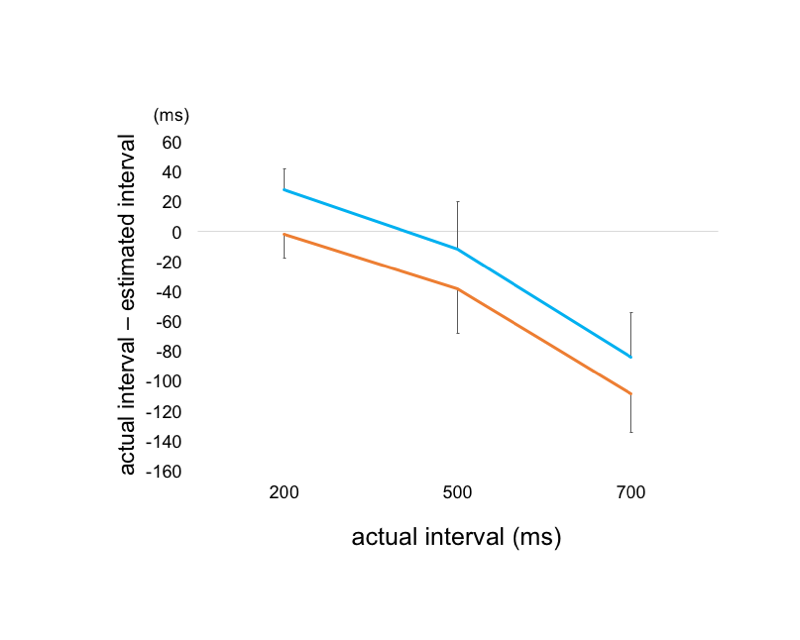

Supplement: Supplemental Information 2 — The blue line represents cluster 1 and the orange line represents cluster 2. Statistical analysis found no interaction between the two clusters and there was no main effect on the intentional binding value. There was no significant difference in intentional binding effect at each interval. Two-way ANOVA was used for statistical analyses. The data represent the means ± standard error. [file peerj-06-6066-s002.png]
